# Automated classification of clinical T staging pulmonary oncology from chest CT reports: a natural language processing framework for real-world data utilization

**DOI:** 10.1186/s12911-026-03596-w

**Published:** 2026-06-17

**Authors:** Nobuo Nishijima, Kento Sugimoto, Shozo Konishi, Shoya Wada, Haruhiko Hirata, Masahiro Yanagawa, Takahiro Tsuboyama, Ayumi Fujii, Taizo Murata, Naoki Mihara, Katsuki Okada, Yasushi Matsumura, Noriyuki Tomiyama, Toshihiro Takeda

**Affiliations:** 1 Department of Medical Informatics, The University of Osaka Graduate School of Medicine, 2-2 Yamadaoka, Suita, Osaka, 565-0871 Japan; 2 Department of Transformative System for Medical Information, The University of Osaka Graduate School of Medicine, 2-2 Yamadaoka, Suita, Osaka, 565-0871 Japan; 3 Department of Respiratory Medicine and Clinical Immunology, The University of Osaka Graduate School of Medicine, 2-2 Yamadaoka, Suita, Osaka, 565-0871 Japan; 4 Department of Diagnostic and Interventional Radiology, The University of Osaka Graduate School of Medicine, 2-2 Yamadaoka, Suita, Osaka, 565-0871 Japan; 5 Department of Medical Informatics, The University of Osaka Hospital, 2-15 Yamadaoka, Suita, Osaka, 565-0871 Japan; 6 National Hospital Organization Osaka National Hospital, Hoenzaka Chuo-ku, Osaka, 2-1-14 Japan

**Keywords:** Natural language processing, Radiology report, TNM

## Abstract

**Background:**

Clinical Tumor, Node, and Metastasis (cTNM) classification is vital for predicting treatment efficacy and prognosis in patients with cancer; however, its utilization in real-world data (RWD) research is hindered by its frequent storage as free text. While chest CT reports include essential information for labeling cT, the extraction process is typically manual and time-consuming.

**Methods:**

We developed a natural language processing (NLP) system capable of labeling clinical T (cT) classifications from chest computed tomography reports. This NLP system employs a combination of deep learning and rule-based algorithms, adhering to the 9th Edition of the TNM Classification for Lung Cancer. The system initially identifies reports containing sufficient information for cT classification and subsequently assigns the appropriate cT substage.

**Results:**

In our training and test sets of 284 and 165 reports, 83% and 85% contained sufficient information for labeling cT substage, respectively. The NLP system demonstrated effective extraction capabilities, achieving weighted F1 scores of 0.96 (0.94–0.98) and 0.93 (0.89–0.97) for the training and test sets, respectively. Among reports accurately predicted to contain adequate information, the cT substage classification achieved weighted F1 scores of 0.97 (0.94–0.99) and 0.95 (0.92–0.99) for the training and test sets, respectively.

**Conclusions:**

This system significantly alleviates annotation costs and enhances the feasibility of utilizing cT data from RWD.

**Supplementary Information:**

The online version contains supplementary material available at 10.1186/s12911-026-03596-w.

## Introduction

Observational research utilizing real-world data (RWD) has realized a marked increase in cancer studies owing to its advantages, including large sample sizes, expedited research timelines, and cost reductions [1]. Accurate cancer staging, which is essential for patient demographic analysis, relies on the Tumor, Node, and Metastasis (TNM) classification system. This system categorizes the extent of cancer to inform treatment planning, prognostic assessment, and treatment efficacy evaluation [2]. The TNM classification comprises the clinical TNM (cTNM) system, which assesses disease extent using clinical data obtained prior to treatment, and the pathological TNM system, which integrates clinical data with findings from surgical and pathological examinations.

A significant challenge in utilizing cTNM classification for cancer research lies in its dependence on unstructured data found in radiology reports. Extracting clinical information from these reports typically requires manual interpretation by specialists according to TNM classification guidelines. This process is time-consuming, and the quality of the extracted data is often inconsistent, heavily reliant on individual expertise. Consequently, developing efficient and reliable methods for extracting this clinical information will facilitate its secondary use [3, 4].

Natural Language Processing (NLP) offers a promising solution for extracting TNM staging information from unstructured reports [5]. In lung cancer, extensive research has focused on the automatic classification of cTNM from chest computed tomography (CT) and positron emission tomography/CT (PET-CT) reports using NLP [6–10]. Traditional rule-based approaches to information extraction enable the development of adaptable rules aligned with staging criteria [6–8]. However, these methods often struggle with generalizability for concept and relation extraction from free-text reports. A Bidirectional Encoder Representations from Transformers (BERT)-based deep learning model has demonstrated favorable performance in entity extraction for cTNM information [9]; nonetheless, comprehensive cTNM classification remains unaddressed [9]. While large language models (LLMs) have also been explored for cTNM classification using chest CT reports [10], their performance, especially in clinical T (cT) classification, has remained suboptimal [10]. Furthermore, explainability becomes critical when deep learning or LLMs handle the entire process from information extraction to cTNM classification [11].

Moreover, radiology reports often contain ambiguous or uncertain descriptions [12]. Such uncertainty complicates the ability to definitively assign a single cTNM classification based solely on report content. Typically, these reports are either manually omitted or quantified during the development of NLP systems for automatic TNM classification, which is impractical [13].

Previously, we developed a deep learning-based NLP system designed to extract clinical information relevant to lung cancer—such as characteristics, size, changes, and anatomical location—from free-text chest CT reports and convert it into a structured format [14]. Additionally, we successfully enhanced our NLP system to classify the certainty of extracted clinical information, accommodating uncertainties expressed in radiology reports [15].

In this research, we aimed to develop an NLP system capable of predicting cT classifications from radiology reports. We proposed that a combined approach utilizing both rule-based and deep learning methodologies would optimize performance, generalizability, and explainability. Additionally, we designed our NLP system to effectively manage radiology reports that are challenging to classify owing to uncertain descriptions or inadequate information. Consequently, a hybrid natural language processing system combining deep learning and rule-based methods was developed to automatically classify free text from Japanese chest CT reports into cT substages. This approach leverages deep learning for flexible structuring of free text while applying rule-based algorithms for cT substage classification in accordance with the 9th Edition of the TNM Classification for Lung Cancer. The significant aspects and objectives of our research were as follows:

- To develop an integrated NLP system combining deep learning and rule-based algorithms for cT substage classification from unstructured chest CT reports, balancing generalizability and explainability.
- To categorize chest CT reports that pose challenges for cT substage labeling owing to uncertainty or insufficient information, automatically classifying them into three categories: (1) reports that can clearly assign a cT substage, (2) reports that suggest potential cT substages despite lacking information or containing uncertain descriptions, and (3) reports that provide no relevant information for cT substage classification.

Initially, we classified chest CT reports into categories (1) to (3) using our NLP system. Subsequently, we classified the cT substage for reports correctly predicted as (1).

## Methods

### Definitions of “definite,” “uncertain,” and “absent” for chest CT reports

To effectively manage radiology reports lacking sufficient information for cT labeling owing to uncertainty or incomplete descriptions, we classified chest CT reports into three categories: “definite” refers to reports that clearly assign a cT substage, “uncertain” refers to reports that suggest potential classifications despite insufficient information or ambiguous descriptions, and “absent” refers to reports that contain no information pertinent to cT substage classification. Each piece of information relevant to cT substage (size, atelectasis, obstructive pneumonia, invasion, lung metastasis, and lymphangitis carcinomatosis) was evaluated for its status as “definite” or “uncertain,” and the final report categorization was determined by integrating this information. The definitions of “definite,” “uncertain,” and “absent” are presented in Table 1, along with example sentences.

**Table 1:** Definitions and example sentences for “definite,” “uncertain,” and “absent”

Classification	Category	Definition	Example
Definite		Reports that clearly assign cT substage	In right S1, a 15-mm solid nodule exists, with lung cancer suspected. In right S8, a 10-mm nodule exists, with lung metastasis suspected.
Uncertain	Uncertain description	Any of the following: Some clinical findings are listed; however, those with a high probability remain unclear. Clinical findings are listed for differentiation. No other diagnosis for differentiation is listed; nevertheless, the diagnosis is uncertain.	In right S1, a 15-mm solid nodule exists, with lung cancer suspected. In right S8, a 10-mm nodule exists. *Both the inflammatory nodule and lung metastasis are equally suspected.*
Uncertain	Unknown location of lung metastasis or lymphangitis carcinomatosis	While the lobe of the lung metastasis was not specifically described, it was indicated in the ipsilateral area against primary lung cancer.	In right S1, a 15-mm solid nodule exists, with lung cancer suspected. *In both lungs, nodules are scattered, with lung metastasis suspected.*
Uncertain	Unknown solid size of part-solid nodule	Overall size of the part-solid nodule is described; however, the diameter of the solid portion is not indicated.	In right S1, *a 15-mm part-solid nodule was detected, with lung cancer suspected.*
Absent		No information for cT substage classification	In right S1, a solid nodule exists, with lung cancer suspected.

## Overview of our NLP system: from information extraction based on unstructured chest CT reports to cT substage classification

**Fig. 1:**
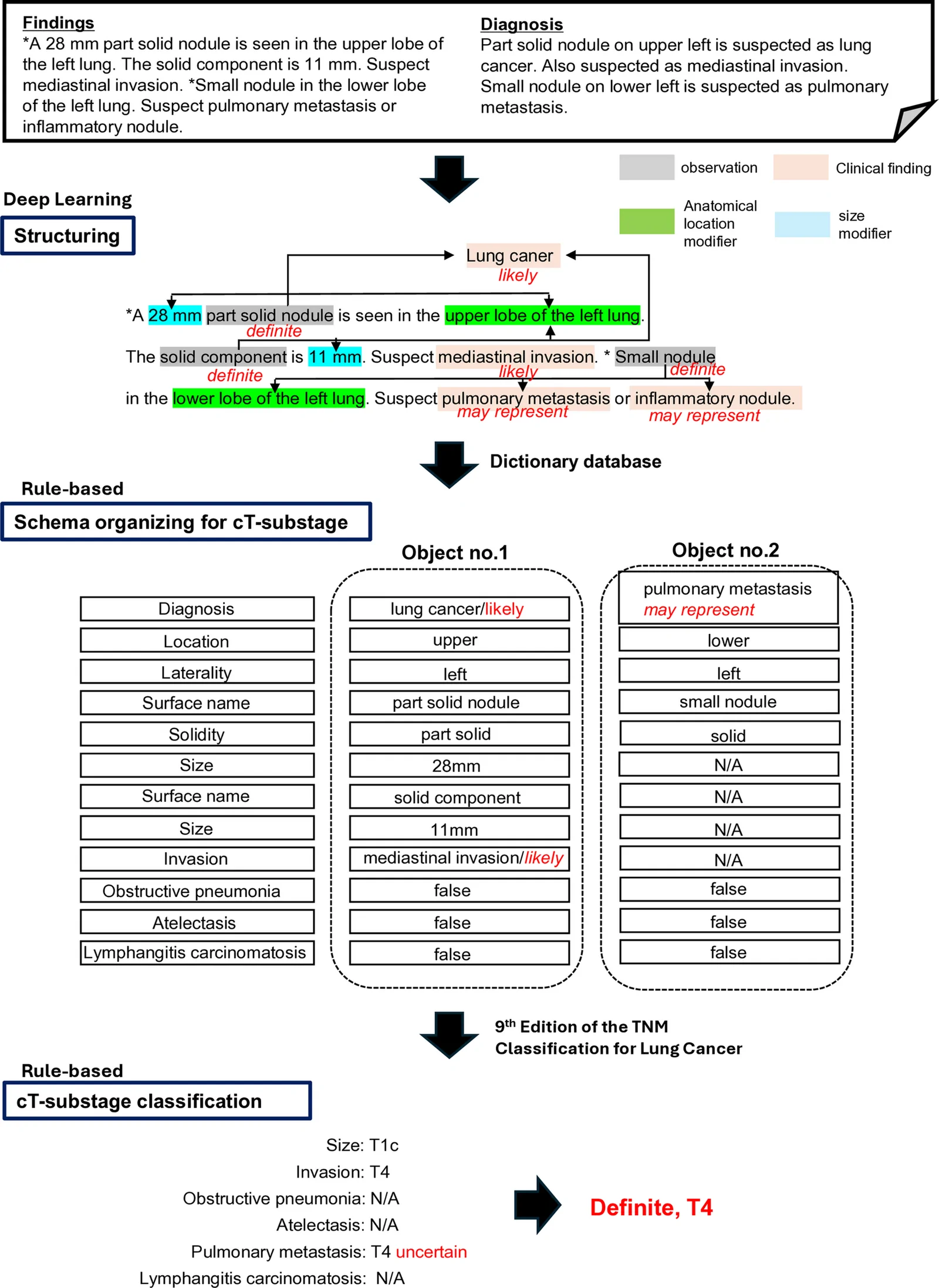
Overview of the developed NLP system. All chest CT reports were written in Japanese; free text examples are in English for clarity

The structure of our NLP system is illustrated in Fig. 1. Our system comprises three steps: 1. deep learning-based “Structuring” (entity extraction and relation definition from unstructured sentences), 2. rule-based “Schema Organizing for cT substage” (dictionary matching for cT substage), and 3. rule-based “cT substage Classification” (outputting cT substage with designations of “definite,” “uncertain,” or “absent”). The rules employed are informed by the 9th Edition of the General Rule for Clinical and Pathological Record of Lung Cancer [3]. Through these steps, we constructed an end-to-end system that takes free text from chest CT reports as input and outputs both the certainty category of the report classified as “definite,” “uncertain,” or “absent” and the corresponding cT substage. 1. “Structuring” was performed using a previously reported deep learning methodology [14, 15]. 2. For “Schema Organizing for cT substage” (dictionary matching for cT substage), we developed new concepts required for cT stage classification. These concepts were linked to the dictionary. In addition, the certainty levels of each concept were output using the same framework defined in our previous research [15]. 3. For “cT substage Classification,” rule-based mappings were constructed to link the new concepts developed in “Schema Organizing for cT substage” to cT stages in accordance with the guidelines [2]. Furthermore, the certainty levels of each concept were mapped to the categories of “definite,” “uncertain,” and “absent.” The details of each step are described below.

### Structuring

To extract entities and define their interactions from unstructured reports, we employed a fine-tuned, pre-trained language model-based information system that we previously developed [14, 15]. This model initially parses sentences and performs word embedding using Mecab [16] and Word2vec [17]. Thereafter, it extracts and classifies entities into three categories: clinical findings, imaging observations, and modifier entities [14]. Modifier entities are further divided into four subcategories: anatomical location, size, change, and characteristics modifiers. Concurrently with entity extraction, certainty scales were assigned to entities related to clinical findings and imaging observations [15]. Certainty is classified into five categories: definite, likely, may represent, unlikely, and denial [15]. Following data extraction and categorization, we defined the relationships among entities. An example of this process can be found in the supplementary materials. Entity extraction utilized a Bidirectional Long Short-Term Memory–Conditional Random Field [18] approach, while certainty and relation definitions were based on BERT [19], informed by performance comparisons with other models in our previous reports [14, 15]. These deep learning processes were executed using a GPU (RTX 8000). Detailed descriptions of entity definitions and the fine-tuning process are documented in previous reports [14, 15].

### Schema organization for cT classification

**Fig. 2:**
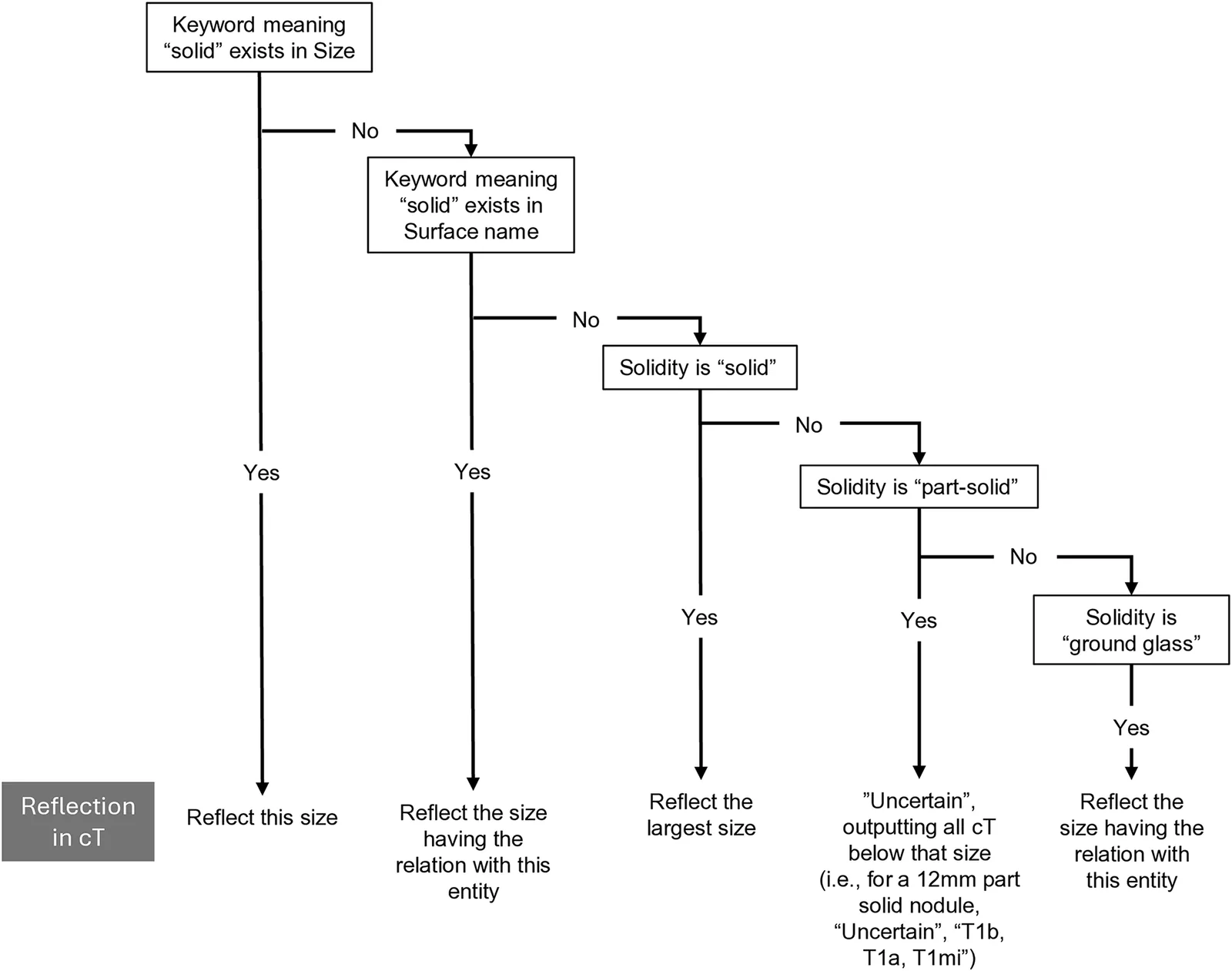
Rule for reflecting size information on clinical T

**Fig. 3:**
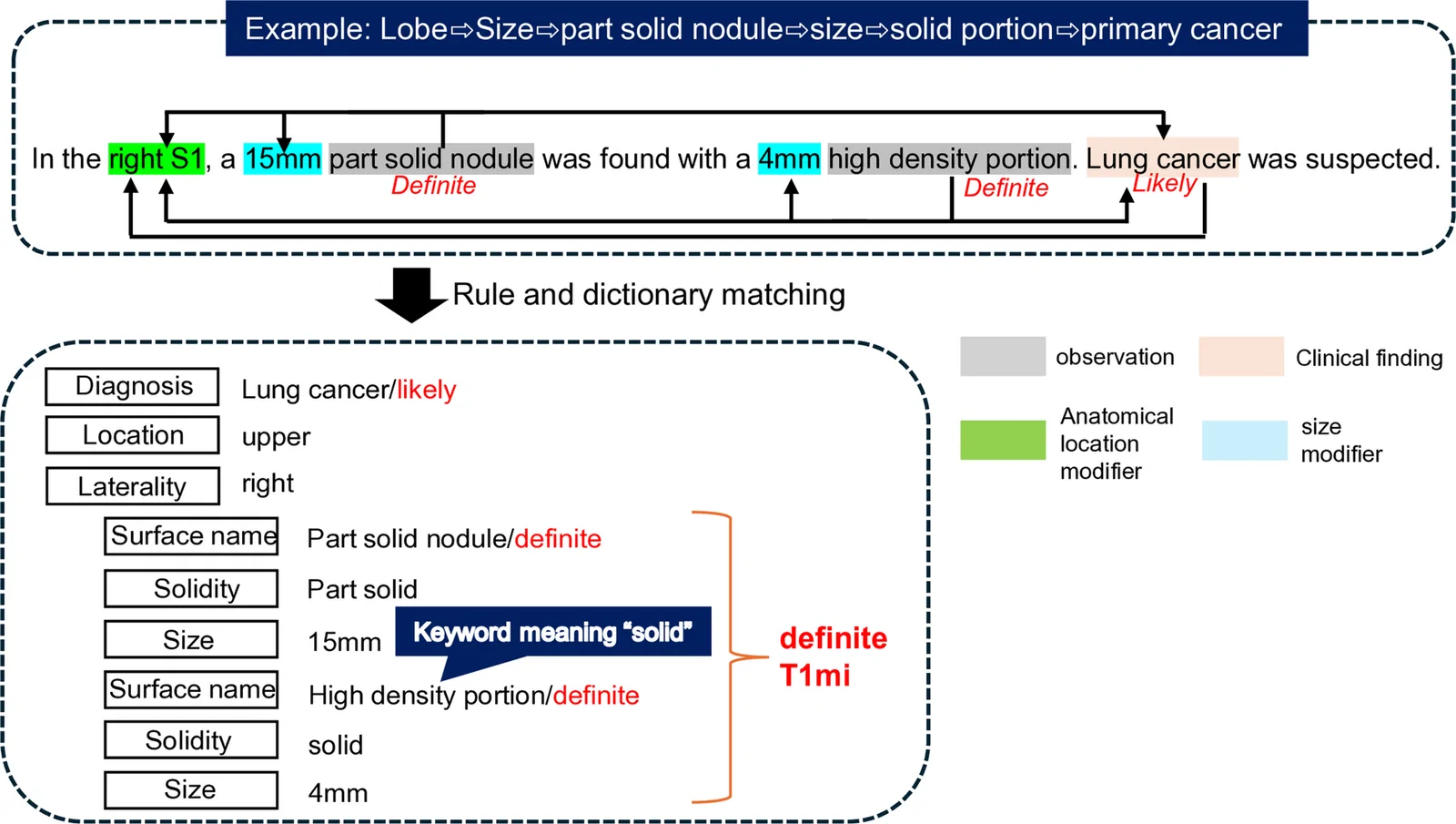
Example of patterns of solidity and size. All chest CT reports were written in Japanese; free text examples are in both English and Japanese for clarity

To address the scarcity of resources for Japanese clinical terms and phrases [20], we utilized a previously constructed dictionary database [21]. This database comprises tables that include surface forms and concepts. The surface table contains phrases and words appearing in the training set reports, while the concept table standardizes these phrases and words. Specific entities extracted from reports—such as imaging observations, clinical findings, anatomical location modifiers, and change modifiers—were string-matched to the dictionary to ascertain their meanings. The dictionary construction process is detailed in a previous article [20]. This dictionary was extended by adding the concept for cT substage, which included information concerning suspected cancer nodules: location, laterality, solidity, total size, solid size, invasions, and the presence or absence of obstructive pneumonia and atelectasis, as well as whether the objective is a primary tumor, lung metastasis, or lung lymphoid carcinomatosis. We established rules to organize the schema for cT substage classification, as the size relevant to cT substage classification varies depending on nodule solidity, total size for solid or ground-glass nodules, and solid size for part-solid nodules. Notably, ground-glass nodules are classified solely as Tis (≤3 cm) or T1a (>3 cm). Therefore, establishing the relationship between observations—such as solid portions and ground-glass opacities for part-solid nodules—and correlating size information with each observation is crucial in this process. Nevertheless, the relationships among observations were not defined during the “structuring” phase of our system [14]. To address this limitation, we developed rules linking nodule solidity (solid, part-solid, and ground-glass) to their respective sizes across various sentences (Fig. 2). We provided examples of sentences containing information on nodules and sizes to construct these rules (Fig. 3, Supplementary Figure 1). Regarding pulmonary metastasis, the laterality and lobe information of satellite nodules concerning the primary tumor had to be organized. Consequently, we formulated rules for lung metastasis based on several sentences, whereby nodules were categorized by associating them with the concepts of “lung cancer” or “pulmonary metastasis” extracted as clinical findings (Supplementary Figure 2). The same rules were applied for lymphangitis carcinomatosis. Invasion, obstructive pneumonia, and atelectasis were organized using relation definitions as described in the structuring process (Fig. 1). Certainty was assigned as outlined in the structuring phase for observations and clinical findings (Fig. 1).

### Clinical T substage classification

An automated rule-based approach was developed to transition organized information into cT substage classification based on each factor, consistent with the 9th Edition of the General Rule for Clinical and Pathological Record of Lung Cancer^2^. The certainty levels were merged from the five categories defined in our previous research [15] into three categories to improve interpretability. “Definite” and “likely” were consolidated into “definite.” To align with the “uncertain” label described in the “Annotation for Sentences in Chest CT Reports,” the certainties of “may represent” and “unlikely” were classified as “uncertain.” If nodule solidity was classified as “part solid” and solid size as “null,” the system returned the cT substage corresponding to the total size and lesser labels, including uncertain labels (e.g., if solidity was “part solid,” solid size “null,” and total size “15 mm,” the system returned “uncertain, T1b, T1a, and T1mi”) (Fig. 3, Supplementary Figure 1). Regarding lung metastasis and lymphangitis carcinomatosis, if their laterality matched that of the primary cancer or was described as “both” while the lobe remained unknown, “uncertain, T3, and T4” was returned (Supplementary Figure 2). The final cT classification was automatically determined using a rule-based approach analogous to the annotation process.

## Data set

**Fig. 4:**
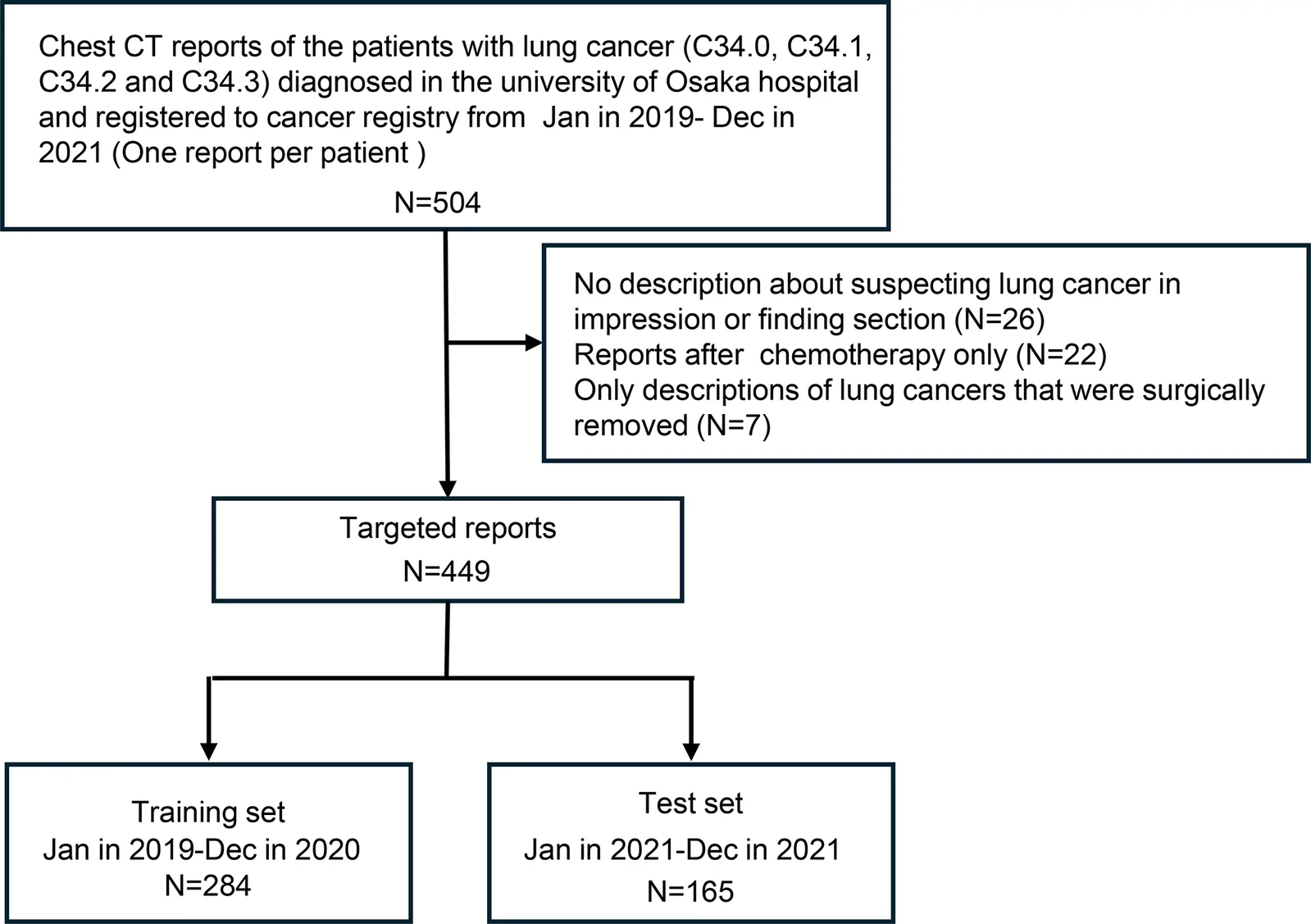
Flow of reports

Chest CT reports of patients diagnosed with lung cancer (C34.0, C34.1, C34.2, and C34.3) at the University of Osaka Hospital, who were registered in the cancer registry in Japan [22], were retrospectively collected (Fig. 4). The reports spanned from January 2019 to December 2021 and comprised multiple sections. We specifically utilized the findings section (which includes clinical objectives and findings) and the diagnosis section (which contains the reporter’s impression related to the findings).

Inclusion criteria for the radiology reports were as follows: the report must contain “suspect lung cancer” in the findings or diagnosis sections and must predate the administration of chemotherapy. Reports solely intended for follow-up after primary lung cancer surgery were excluded. Because the primary objective of this study was to develop an NLP system for cT classification, we selected one report per patient, opting for the last report prior to surgery or the initiation of treatment among the reports that satisfied the selection criteria to maximize informative content. If the last report before surgery or treatment lacked sufficient information corresponding to uncertain or absent, an alternative report containing the necessary information was selected. The dates of the original and substituted reports, cT substage, and the interval in days between the two reports are provided in Supplementary Table 2. The reports were subsequently divided into training (January 2019 to December 2020) and test (January 2021 to December 2021) sets (Fig. 4).

A total of 284 chest CT reports were included in the training sets, which were utilized for algorithm development and optimization. For the test sets, 165 chest CT reports were employed, which were not used in algorithm development or refinement.

For NLP, unstructured sentences from the “findings” and “diagnosis” sections underwent a cleaning process.

## Annotation of sentences in chest CT reports

Annotation was conducted by reviewing the findings and diagnosis sections of chest CT reports in accordance with the 9^th^ Edition of the General Rule for Clinical and Pathological Record of Lung Cancer^2^. As outlined in the “Definitions of ‘definite,’ ‘uncertain,’ and ‘absent’ for chest CT reports” section, annotation was performed based on sentences corresponding to the definitions in Table 1. Examples of annotation are detailed in Supplementary Table 1. If a report lacked information for any element pertinent to cT substage classification, “absent” was assigned for that element. Ultimately, each report was annotated for each component of the cT substage (size, atelectasis, obstructive pneumonia, invasion, lung metastasis, and lymphangitis carcinomatosis) using 19 classifications (Tis, T1mi, T1a, T1b, T1c, T2a, T2b, T3, T4, Tis uncertain, T1mi uncertain, T1a uncertain, T1b uncertain, T1c uncertain, T2a uncertain, T2b uncertain, T3 uncertain, T4 uncertain, and absent). Thereafter, we assigned a final classification of “definite,” “uncertain,” or “absent” and determined the final cT substage for each report by integrating all information according to the rules outlined in Supplementary Fig. 1. Examples of annotations are displayed in Supplementary Table 1. The training set was annotated by a single non-physician researcher, while the test set was annotated by two physicians. The non-physician researcher annotated the training set under the supervision of physician researchers. In cases where the cT classification differed between the two annotators regarding the test set, a gold standard was established through discussion with a third party. Through this collaboration, one annotation result that both annotators agreed upon was modified as necessary. Inter-annotator agreement (IAA) was calculated using Cohen’s kappa [23], a widely employed metric. The mean IAA score for the two annotators regarding the exact match of final cT classifications in the test sets was 0.91, indicating a nearly perfect level of agreement [24].

## Statistical analysis

Accuracy was defined as the proportion of correct classifications for each criterion. The performance of both the training and test sets was evaluated using precision, recall, and F1 scores. For overall performance calculations, the precision, recall, and F1 scores of each subcategory were weighted according to the dataset size. The performance of classification into “definite,” “uncertain,” and “absent” was assessed using the entire dataset (training: *n* = 284, test: *n* = 165). The performance of cT substage classification was evaluated among subsets that had been correctly predicted as “definite.” To evaluate performance as an end-to-end system, the performance of cT substage classification for “uncertain” and “absent” was also evaluated. Ninety-five percent confidence intervals for F1 scores were estimated using non-parametric bootstrap resampling at the case level (2,000 samples). Weighted averages were calculated based on the number of ground-truth cases per class.

A confusion matrix was created for both the training and test sets to illustrate the performance of the “definite,” “uncertain,” and “absent” classifications.

Error analyses were conducted focusing on two specific cases: 1. false positives incorrectly classified as “definite” during the classification task into “uncertain” or “absent,” and 2. instances where the cT substage was misclassified among those accurately predicted as “definite.” The rationale for concentrating on these two error types is that such misclassifications may go unnoticed in real-world applications of the system. In the former analysis, errors were categorized and subcategorized based on “uncertain type” and “source of error.” In the latter analysis, errors were categorized and subcategorized according to “cause of error” and “source of error.” In both analyses, “source of error” referred to the point of error within our NLP overview (Fig. 1).

## Results

**Fig. 5:**
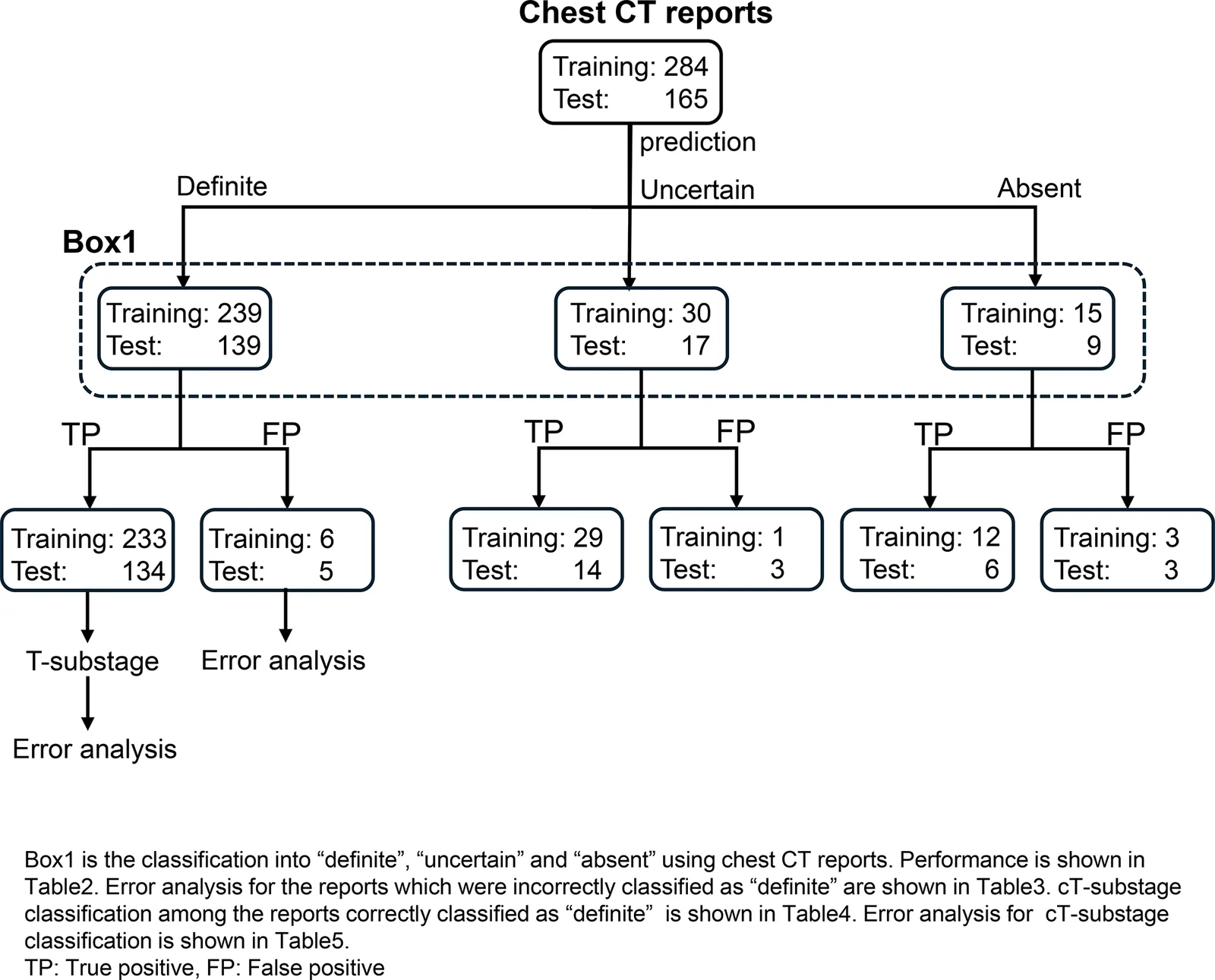
Overview of results. Box 1 displays classification into “definite,” “uncertain,” and “absent” using chest CT reports. Performance is detailed in Table 2. Error analysis for reports misclassified as “definite” is presented in Table 3. cT substage classification among reports correctly classified as “definite” is shown in Table 4. Error analysis for cT substage classification is in Table 5. TP: true positive, FP: false positive

An overview of the results from our tasks is presented in Fig. 5.

**Fig. 6:**
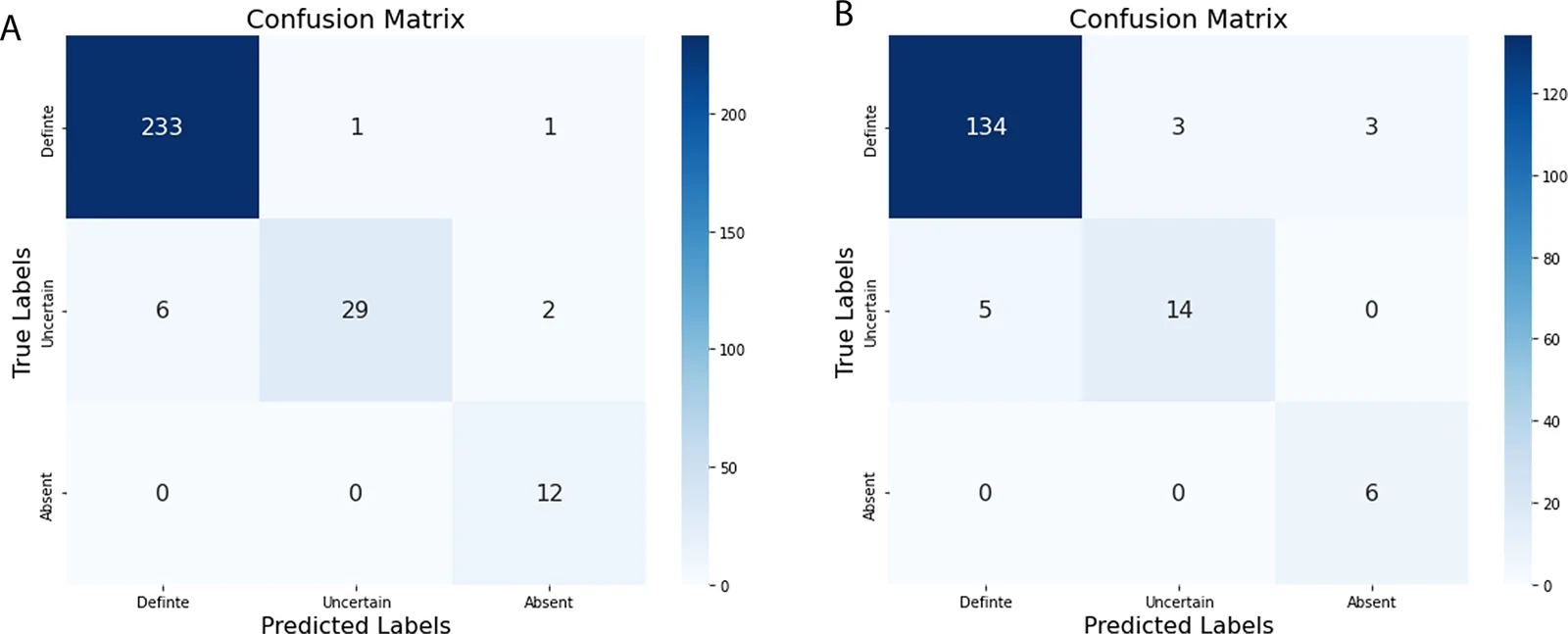
Confusion matrix for “definite,” “uncertain,” and “absent”

Accuracy was determined to be 0.96 and 0.93 for the training and test sets, respectively. Predictions of “definite,” “uncertain,” and “absent” classifications are detailed in Table 2. The overall weighted F1 scores for the training and test sets demonstrated strong performance. The recall score for “uncertain” was notably lower than that for other classifications in both training and testing phases. Additionally, precision for “absent” was lower than that for the other classifications in both phases. The confusion matrix is depicted in Fig. 6.

**Table 2:** Classification into “definite,” “uncertain,” and “absent”

	Number (％)	Precision	Recall	F1 score
**Training**				
Definite	235 (83)	0.97	0.99	0.98 (0.97–0.99)
Uncertain	37 (13)	0.97	0.78	0.87 (0.77–0.94)
Absent	12(4)	0.80	1.00	0.89 (0.71–1.00)
Overall/Weighted average	284 (100)	0.97	0.96	0.96 (0.94–0.98)
**Test**				
Definite	140 (85)	0.96	0.96	0.96 (0.94–0.98)
Uncertain	19 (12)	0.82	0.74	0.78 (0.58–0.91)
Absent	6 (4)	0.67	1.00	0.80 (0.50–1.00)
Overall/Weighted average	165 (100)	0.94	0.93	0.93 (0.89–0.97)

We performed an error analysis on cases predicted as “definite” despite being annotated as “uncertain,” as such errors are critical in real-world implementations. Out of 235 training cases and 140 test cases, 6 and 5 were identified as errors, respectively (Table 3). The most common types of “uncertain” errors in the training and test sets included a lack of solid size despite the existence of a part-solid nodule (*n* = 2 for each set) and ambiguity regarding the lobe involved in pulmonary metastasis or lymphoid carcinomatosis (*n* = 2 for each set).

**Table 3:** Error analysis of classification into “definite,” “uncertain,” and “absent”

Uncertain type	Source of error	Detail	Training (n = 6)	Test (n = 5)
No solid size information about part-solid nodule	Schema organizing for cT substage	Although the overall diameter is recorded for part-solid nodules, no record of the solid portion exists. The overall diameter is reflected as the size of the solid or ground-glass portion.	2 (T1a, T1mi) ⇨ Tis (T1c, T1b) ⇨ T1b	2 (T1b,T1a,T1mi)⇨ T1b (T1c, T1b, T1a, T1mi) ⇨ Tis
Unclear location of metastasis and lymphangitis carcinomatosis against the primary tumor	Schema organizing for cT substage	Although the lobe of cancerous lymphangitis was described, that of the primary tumor was not.		1 （T3, T4) ⇨ T4
	Schema organizing for cT substage	Based on the description, “There is a 4-cm solid nodule in the right upper lobe, with small nodules scattered across both lungs, suggesting lung cancer and multiple lung metastases,” the positions between the primary tumor and lung metastasis could not be classified.	2 （T3, T4) ⇨ T2a (T3, T1b) ⇨ T1b	1 （T3, T4) ⇨ T1c
No clear description of “tumor” related to the nodule	Schema organizing for cT substage	“Tumor” description was confined to the diagnosis section. Limitations exist in linking the primary tumor mentioned in the diagnosis to the nodule discussed in the findings.	1 （T3, T4) ⇨ T1c	
Certainty	Structuring	The expression, “There is a suspicion of infiltration, but it is unclear,” was classified as “definite.”	1 （T2a, T4) ⇨ T2a	
Final classification rule	cT substage classification	An error occurred in the rules for deriving the final output from the classifier.		1 （T2a, T4) ⇨ T2a

**Fig. 7:**
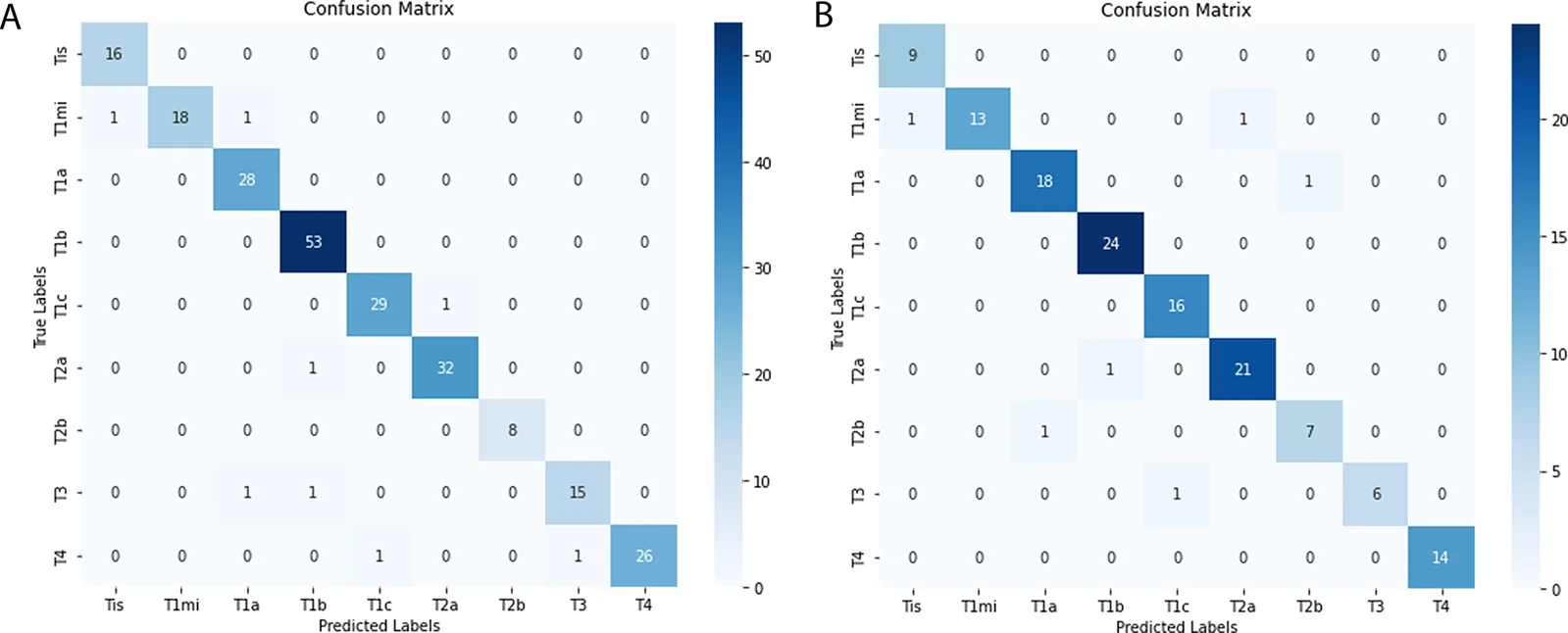
Confusion matrix for T substage classification among reports correctly classified as “definite”

The accuracy of cT substage classification among subsets that had been correctly predicted as “definite” was 0.97 and 0.96 for the training and test sets, respectively. The performance of cT substage classification indicated that the weighted F1 score exceeded 0.95 (0.92–0.99) in the test set, with each individual F1 score also reflecting favorable performance (Table 4). The corresponding confusion matrix is presented in Fig. 7.

**Table 4:** cT substage classification among true positives classified as “definite”

	Number (％)	Precision	Recall	F1 score
**Training**				
Tis	16 (7)	0.94	1.00	0.97 (0.89–1.00)
T1mi	20 (9)	1.00	0.90	0.95 (0.86–1.00)
T1a	28 (12)	0.93	1.00	0.97 (0.91–1.00)
T1b	53 (23)	0.96	1.00	0.98 (0.95–1.00)
T1c	30 (13)	0.97	0.97	0.97 (0.92–1.00)
T2a	33 (14)	0.97	0.97	0.97 (0.92–1.00)
T2b	8 (3)	1.00	1.00	0.97 (0.92–1.00)
T3	17 (7)	0.94	0.88	0.98 (0.95–1.00)
T4	28 (12)	1.00	0.93	0.96 (0.90–1.00)
Overall/ Weighted average	233 (100)	0.97	0.97	0.97 (0.94–0.99)
**Test**				
Tis	9 (7)	0.90	1.00	0.95 (0.80–1.00)
T1mi	15 (11)	1.0	0.87	0.93 (0.80–1.00)
T1a	19 (14)	0.95	0.95	0.95 (0.85–1.00)
T1b	24 (18)	0.96	1.00	0.98 (0.93–1.00)
T1c	16 (12)	0.94	1.00	0.97 (0.89–1.00)
T2a	22 (16)	0.95	0.95	0.95 (0.87–1.00)
T2b	8 (6)	0.88	0.88	0.88 (0.62–1.00)
T3	7 (5)	1.00	0.86	0.92 (0.67–1.00)
T4	14 (10)	14 (10)	1.00	1.00 (1.00–1.00)
Overall/ Weighted average	134 (100)	0.96	0.95	0.95 (0.92–0.99)

An error analysis was conducted for the classification of cases annotated as “definite” that were also accurately predicted as “definite” (Table 5). The most frequent causes of errors in the training and test sets included improper classification of the solidity or overall diameter of ground-glass nodules (*n* = 2 for each set), a considerable distance between the primary cancer and the solid nodule (*n* = 2 and *n* = 1, respectively), and mispositioning between the primary cancer and pulmonary metastasis or lymphoid carcinomatosis (*n* = 2 and *n* = 1, respectively).

**Table 5:** Error analysis of T substage classification among true positives classified as “definite”

Type of error	Source of error	D	Training (n = 8)	Test (n = 6)
Misclassification of size	Schema organizing for cT substage	The overall size was considered the solid size in part-solid nodules, or the solid or part-solid nodule was incorrectly classified as “ground glass.”	2 T1mi ⇨ T1a T1mi ⇨ Tis	2 T1a ⇨ T2b T1mi ⇨ Tis
Sizable distance between clinical finding and objectives.	Schema organizing for cT substage	Description regarding “tumor” was limited to the diagnosis section, leading to challenges in linking the primary tumor to the nodule.	2 T3 ⇨ T1b T2a ⇨ T1b	
	Structuring	The relationship between the nodule (observation) and primary tumor (clinical finding) was not defined owing to the considerable distance resulting from other text and line breaks inserted between them.		1 T2b ⇨ T1a
Misclassification of lung metastasis or cancerous lymphangitis	Schema organizing for cT substage	When multiple metastatic sites were present or when the lobe was not clearly specified, such as “near the primary tumor,” the rules did not adequately define the relationship among the primary tumor, metastatic sites, and cancerous lymphangitis.	2 T3 ⇨ T1a T4 ⇨ T1c	1 T3 ⇨ T1c
Expression outside of the dictionary	Schema organizing for cT substage	Terms like “mediastinal side” and “right pleura” were not recognized correctly, leading to misclassification.	1 T4 ⇨ T3	1 T2a ⇨ T1b
Lung atelectasis	Schema organizing for cT substage	Atelectasis not corresponding to T2a was incorrectly classified as T2a.	1 T1c ⇨ T2a	1 T2a ⇨ T1b

The accuracy of cT substage, “uncertain,” and “absent” was determined to be 0.94 (training) and 0.90 (test). The weighted F1 score for this classification was 0.90 (0.85–0.94) in the test set (Supplementary Table 3).

Error analysis focusing on dictionary matching was conducted. Among the 165 cases in the test set, four classification errors were attributable to failures in dictionary matching. Detailed are summarized in Supplementary Table 4.

## Discussion

### Error analysis

Our system achieved an F1 score of 0.96 for “definite” classifications in the test set. Given that over 80% of our dataset was annotated as “definite,” this performance is commendable. The F1 score for “uncertain” classifications was 0.78 in the test set, attributed to a lower recall of 0.74. This suggests that the system may overlook some true “uncertain” cases. These errors were primarily caused by two incidents. First, classification errors arose in reports lacking solid part size in part-solid nodules (Table 3). In these instances, misclassification occurred, remaining within the T1 category (e.g., [T1b, T1a, and T1mi] ⇨ [T1b]) or from T1 to Tis (e.g., [T1a and T1mi] ⇨ [Tis]). Considering that T1 or Tis corresponds to stage 0 or 1 when N and M are N0 and M0, respectively, these errors may not be critical. Second, “uncertain” reports that ambiguously described the lobe of lung metastasis or lymphoid carcinomatosis were misclassified as “definite” (Table 3). These misclassifications typically transitioned from higher cT to lower cT (e.g., [T3 and T4] ⇨ [T1c]), which could be significant because severe stages may be overlooked owing to stage disparity. Such reports often contained phrases like “small nodules were scattered in both lungs, with pulmonary metastases suspected,” implying that “pulmonary metastasis occurred alongside the primary cancer” rather than providing specific lobe information. If metastasis occurs in the ipsilateral lobe, it is categorized as M1a, which is classified as stage 4, independent of cT classification. Consequently, the impact of this type of error may be less significant if the system expands to include M classification.

The classification of cT substage using cases correctly predicted as “definite” exhibited excellent performance (Table 4). Notably, the weighted F1 score reached 0.95 in the test set, with accuracy, precision, recall, and F1 scores for each cT classification all exceeding 0.86. The most common cause of error is related to the classification based on size information (Table 5). Size is critically important for cT classification. Prior research indicates that the performance of cT classification declines when focusing solely on size-based classification [6]. Furthermore, the “9^th^ edition of the General Rule for Clinical and Pathological Record of Lung Cancer” necessitates the separation of total and solid size, complicating classification tasks. To the best of our knowledge, our research is the first to incorporate nodule solidity and differentiate between solid and total size in NLP-based cT classification. In the test set detailed in Table 4, 62% (83/134) of reports were annotated with Tis, T1mi, T1a, T1b, or T1c based exclusively on size information. Considering that only two errors occurred related to size information, our system demonstrated effective classification compared with simple rule-based approaches [6–8]. Error analysis revealed that reports with considerable distance between primary cancer and solid nodule lacked defined relationships, resulting in three errors in training and one in testing. Considering that this error did not increase in the test set, it is likely non-critical and may improve with a larger dataset. In cases where multiple nodules were associated with lung metastasis or the lung metastasis site was vaguely described, the relationship between the primary cancer location and lung metastasis was inadequately defined. In this study, relationships between clinical findings and observations were established through deep learning; however, those between clinical findings (e.g., primary lung cancer and lung metastasis) were defined using rule-based methods. One barrier to applying deep learning effectively is the assumption that the primary cancer and lung metastasis are too distant within the text for accurate identification. Notwithstanding, Wang et al. proposed a BERT-based method to circumvent this limitation [25]. Accordingly, utilizing deep learning for defining relationships between clinical findings, akin to the relationship definition between clinical findings and observations, presents a promising solution.

### Take-home messages

We developed a hybrid NLP system integrating deep learning and rule-based methods to classify cT substages in chest CT reports. Our system effectively classifies reports as “definite,” “uncertain,” or “absent” with commendable performance, particularly in cT substage classification for reports accurately predicted as “definite.” Our study has two key messages.

First, our work demonstrates the utility of a hybrid approach that combines deep learning with rule-based methods. Supplementary Table 5 illustrates the performance of cT classification for each method. Matsuo et al. indicated that LLMs did not perform well in cT classification, yielding an accuracy of 0.44 for chest CT reports [10]. Meanwhile, Nobel et al. reported that a rule-based system achieved an accuracy of 0.87 with their test set [8]. Although rule-based systems may exhibit limited generalizability owing to their inflexibility, the classification of cT inherently operates within a rule-based framework, applying clinical information related to primary lung cancer to cancer staging [2]. Our rule-based approach may partially address the generalizability limitations of such systems by leveraging deep learning for entity extraction and relationship definition from unstructured reports. Additionally, the partial rule-based method offers advantages for cT classification, as the system is continually updated based on accumulating evidence. Our approach can easily adapt to new guidelines by modifying the rules. We initiated our research using the 8^th^ edition of the official lung cancer guidelines, updating them throughout the research period.

Second, radiology reports often contain various uncertain or ambiguous phrases. Callen et al. discovered that 55.0% of thoracic radiology reports included uncertainty-related terminology [12]. Therefore, effectively addressing these uncertain or ambiguous reports is crucial for the application of NLP in the secondary usage of radiology reports. Nevertheless, previous research has either omitted or quantified reports containing such descriptions in the development of NLP systems aimed at automatically classifying TNM from radiology reports, with the exception of Barlow’s study [13]. Barlow et al. reported an F1 score of 0.87 in detecting uncertain and ambiguous PET-CT reports within an internal test set utilizing pretrained transformer-based language models [13]. While the performance of this approach was excellent, the authors recognized the requirement for further refinement of their system. Our system offers three distinct advantages. First, we incorporated a rule-based approach, enabling comprehensive error analysis. This hybrid deep learning and rule-based system facilitates error analysis, leading to effective system refinement and enhanced explainability. Second, Barlow et al. exclusively focused on determining whether reports contained ambiguous or uncertain expressions. Nonetheless, assigning a cTNM classification based on other clearly stated information within the same report may be possible, despite the presence of ambiguous terminology. For instance, regarding cT substage, T4 could be labeled based on the statement, “The size of the nodule in right S8 exceeds 8 cm. A small nodule exists in S8, but it is difficult to determine whether it is an inflammatory nodule or a lung metastasis.” Therefore, we classified the reports as “definite,” “uncertain,” or “absent” according to an annotation guideline based on whether the report provided clear information for cT substage labeling, adopting a more practical approach by verifying each sentence individually. Third, our system exhibited effective cT substage labeling in reports correctly classified as “definite,” achieving excellent performance.

### Expected clinical deployment of our system

Our system achieved an F1 score of 0.96 for predicting “definite” classifications and an F1 score of 0.95 for cT substage among reports accurately predicted as “definite” in the test set. Furthermore, an F1 score of 0.9 was also achieved on the test set for the end-to-end 11 class classification of cT substage, “uncertain”, and “absent”. Given these results, we may employ the output of cT substage classification in real-world applications, potentially mitigating the time-consuming burden of manual annotation. Additionally, our NLP system may indirectly enhance real-world clinical practice by minimizing the number of radiology reports that are challenging to classify owing to ambiguous descriptions or insufficient information. Our dataset primarily contains two types of “uncertain” reports. First, certain reports lack sufficient information for cT classification, such as those that only provide the total size of a part-solid nodule or fail to specify the position of a satellite nodule relative to the primary cancer. These reports account for 51.4% (19/37) and 68.4% (13/19) of the training and test sets annotated as “uncertain,” respectively. Second, radiologists have found it challenging to label cT based on chest CT, particularly in cases where invasion is difficult to confirm or where it is unclear whether a nodule represents lung metastasis or an inflammatory process. These reports account for 48.6% (18/37) and 31.6% (6/19) of the training and test sets annotated as “uncertain,” respectively. Particularly for the first category, our system’s prevalence among radiologists may contribute to an increase in the frequency of radiology reports capable of labeling cT substage. Furthermore, by labeling cT substage for the radiology images corresponding to reports predicted as “definite,” our NLP system may aid in constructing extensive datasets of radiology images with cT labeling, thus advancing the development of deep learning models that can predict cT substage from these images.

## Limitations and future directions

This research bears certain limitations. First, only chest CT reports were analyzed. Although chest CT is a suitable modality for labeling cT in lung cancer, our system warrants validation with other modalities, such as PET-CT, as well as the development of similar systems for N and M classifications. Second, the annotation was performed in accordance with the 9th edition of the Lung Cancer Treatment Protocol; however, the chest CT reports were sourced from the period between 2019 and 2021, during the era of the 8th edition. Nevertheless, no significant alterations in T classification occurred between the 8th and 9th editions, which is not expected to affect radiologists’ descriptions. Third, we established selection criteria to extract reports suitable for cT labeling, focusing on developing an NLP system that accurately classifies cT labels. For real-world implementation, verifying results even without predetermined selection criteria is imperative. Fourth, this study was conducted using data from a single institution, and reports from external institutions were not included. Therefore, external validation using independent cohorts is necessary. Although our deep learning-based methods—both with and without dictionary support—have previously been validated using datasets from external institutions [14, 20], further validation of the complete system is warranted. Fifth, the present system was developed using Japanese chest CT reports and is not directly applicable to reports written in English. Nevertheless, the underlying methodology, which combines deep learning with a rule-based framework, may be transferable to other languages, as cT classification inherently follows rule-based clinical guidelines. Sixth, an imbalance emerged in the distribution of cT stages, with fewer than 10 cases in some cT categories. However, because the cT classification is predominantly rule-based, the impact of this class imbalance is presumably limited. Seventh, the training set was annotated by a single non-physician researcher, while the test set was annotated by two physicians. Even though the non-physician researcher annotated under the supervision of a physician and the IAA of the test set was favorable, this asymmetric annotation represents a potential source of bias.

## Conclusions

We successfully developed a system capable of detecting reports that are sufficiently informative for labeling and accurately predicting cT substage for these informative reports. This system potentially addresses the barriers to utilizing cT from RWD by alleviating annotation costs while maintaining excellent performance. Future research should focus on extending this system to other modalities and N/M classifications.

## Electronic supplementary material

Below is the link to the electronic supplementary material.

Supplementary material 1

## Data Availability

The datasets used and analysed during the current study are available from the corresponding author on reasonable request.
